# Role of Vaginal Microbiota and Oral *Lactobacillus* Supplementation in Recurrent Urinary Tract Infections of Menopausal Women: Protocol for the VaMirUTI Cohort Study

**DOI:** 10.3390/bioengineering12111134

**Published:** 2025-10-22

**Authors:** Dimitri Barski, Patrick Finzer, Klaus Golka, Olga Renner, Ralph Wirtz, Thorsten Ecke, Thomas Otto

**Affiliations:** 1 Department of Urology, Rheinland Klinikum Neuss, Preussenstr. 84, 41464 Neuss, Germany; 2 German Microbiome Prevention Group (GMPG), Behringstr. 2, 41464 Neuss, Germany; 3 Department of Microbiology, University of Düsseldorf, Moorestr. 5, 40225 Düsseldorf, Germany; 4 Department of Urology, Charité—Universitätsmedizin Berlin, Corporate Member of Freie Universität Berlin, Humboldt-Universität zu Berlin, and Berlin Institute of Health, Charitéplatz 1, 10098 Berlin, Germany; 5 Department of Urology, Helios Hospital, Pieskower Str. 33, 15526 Bad Saarow, Germany

**Keywords:** vaginal microbiota, urinary tract infection, probiotics, *Lactobacillus*, postmenopause, cohort study, vaginal estrogen, 16S rRNA sequencing

## Abstract

(1) Background: Recurrent urinary tract infections (rUTIs) are common among peri- and postmenopausal women, partly due to hormonal changes that disrupt the vaginal microbiota. A reduction in *Lactobacillus* dominance is associated with increased risk of rUTI. Although antibiotics remain the standard of care, their use contributes to the emergence of multidrug-resistant pathogens. Probiotics may offer a non-antibiotic alternative; however, clinical evidence remains limited. (2) Methods: The VaMirUTI study is a prospective, monocentric, non-randomized cohort investigating the combined effect of oral probiotics and vaginal estriol on vaginal and urinary microbiota in peri- and postmenopausal women with recurrent UTIs. The primary endpoints are (i) change in *Lactobacillus* dominance at 3 months and (ii) UTI recurrence at 12 months. A total of 100 women (70 rUTI, 30 controls) will be followed for up to 12 months. Vaginal swabs and urine samples will be collected at baseline, during UTI episodes, and at study completion. Vaginal microbiota composition will be analyzed by 16S rRNA gene sequencing. (3) Results: This protocol outlines the study design and methodology. The primary outcome is the change in vaginal *Lactobacillus* dominance following the intervention. Secondary outcomes include UTI recurrence rates and the identification of microbiota signatures associated with rUTI. (4) Conclusions: The VaMirUTI study will clarify the relationship between vaginal microbiota, oral probiotic supplementation, and rUTI in menopausal women, potentially informing future non-antibiotic preventive strategies.

## 1. Introduction

Urinary tract infections (UTIs) are among the most prevalent bacterial infections worldwide, particularly affecting women. More than half of all women experience at least one UTI during their lifetime, and approximately 30–44% develop recurrent episodes, termed recurrent urinary tract infections (rUTIs) [1,2]. rUTIs are clinically defined as three or more infections within a 12-month period or two or more within six months [3].

Menopausal and postmenopausal women represent a particularly vulnerable population, primarily due to estrogen deficiency associated with menopause. This hormonal change leads to physiological alterations in the urogenital tract, including thinning of the epithelium, decreased glycogen production, and shifts in the composition and stability of the vaginal microbiota [2,4,5]. In healthy, reproductive-age women, the vaginal microbiota is predominantly composed of *Lactobacillus* species such as *L. crispatus*, *L. jensenii*, *L. gasseri*, and *L. iners*. These species help maintain a low vaginal pH through the production of lactic acid and antimicrobial substances like hydrogen peroxide [6], creating an acidic environment that inhibits pathogen growth of pathogenic bacteria. However, estrogen withdrawal during menopause leads to a loss of *Lactobacillus* dominance, facilitating colonization by uropathogenic bacteria such as *Escherichia coli* [2,3,4,5,6,7]. The pathophysiology of rUTIs involves complex interactions between host factors, uropathogens, and the urogenital microbiome.

There is consistent evidence that topical vaginal estrogen promotes *Lactobacillus* recovery and lowers pH, while probiotic effects are strain- and route-dependent. Intravaginal estrogen is more directly effective, while oral estrogen can modulate vaginal flora in some studies [8,9]. Although antibiotic therapy remains the standard treatment, long-term or repeated use results in major drawbacks, including the emergence of multidrug-resistant pathogens, disruption of the host microbiome, and side effects such as *Clostridioides difficile* infection [10]. Consequently, non-antibiotic preventive strategies are gaining attention. Among these, topical vaginal estrogen therapy has been shown to restore the urogenital epithelium and support recolonization with beneficial *lactobacilli* [7]. Oral probiotic supplementation, particularly with specific *Lactobacillus* strains, is another promising approach. It is hypothesized that oral probiotics may influence not only the gut microbiota but also exert systemic and local effects on the vaginal and urinary microbiota. *Lactobacillus crispatus* and *Lactobacillus rhamnosus* have shown potential in modulating the vaginal microbiota and reducing UTI recurrence [11,12]. Nonetheless, evidence supporting their routine clinical use remains limited and inconsistent.

Feasibility and small trials suggest combined topical estrogen and probiotics are safe and may be better than either alone in restoring a *Lactobacillus*-dominant vaginal microbiota, but RCT evidence remains limited. Recent pilot/feasibility trials report safety and suggestive benefit but are underpowered for definitive clinical endpoints [13,14].

The VaMirUTI (**Va**ginal **Mi**crobiota in **R**ecurrent **U**rinary **T**ract **I**nfections) cohort study is designed to evaluate the effects of combined prophylaxis—oral *Lactobacillus* and vaginal estriol—on vaginal microbiota composition and rUTI recurrence over a 12-month follow-up. By comparing microbiota profiles before and after intervention, this study aims to identify microbial patterns predictive of treatment response and recurrence risk.

This protocol follows MDPI guidelines and provides sufficient methodological detail to ensure reproducibility.

## 2. Materials and Methods

### 2.1. Study Design and Objectives

The VaMirUTI study is a prospective, single-center cohort study conducted at the Department of Urology, Rheinland Klinikum Neuss, in collaboration with the Department of Microbiology, University of Düsseldorf. This study will enroll peri- and postmenopausal women with recurrent urinary tract infections (rUTIs) and age-matched healthy postmenopausal controls. Participants will be followed for up to 12 months, with assessments and biological sample collections at baseline, during follow-up, and upon new UTI episodes.

The primary objectives are to

(i) Evaluate the effects of combined oral *Lactobacillus* supplementation and vaginal estrogen therapy on the vaginal and urinary microbiota;
(ii) Determine UTI recurrence rates during follow-up.

The secondary objectives include identifying microbial and clinical predictors of treatment response, assessing quality of life (QoL) and menopausal symptoms, and evaluating the safety and tolerability of the intervention.

### 2.2. Participants

Women ≥ 40 years who are peri- or postmenopausal are eligible for inclusion.

Inclusion criteria:

- rUTI group: ≥3 culture-confirmed UTIs in the past 12 months or ≥2 in the past 6 months. Control group: no UTI within the past 12 months and no antibiotic use in the preceding 4 weeks.

Exclusion criteria:

- Structural or functional abnormalities of the urinary tract;
- Systemic infections or immunosuppression;
- Recent antibiotic use (<4 weeks);
- Known allergy to probiotics or estriol contraindications (e.g., history of estrogen-dependent tumors, thromboembolism, unexplained bleeding).

Sample size calculation:

Sample size (n = 100) provides 80% power (α = 0.05) to detect a 25% increase in *Lactobacillus*-dominant community state type (CST) prevalence (30%→55%) and a 30% reduction in recurrence rate at 12 months, assuming 15% loss of participants over time in a study.

### 2.3. Intervention

Participants in the rUTI group will receive one oral probiotic capsule daily and low-dose vaginal estriol cream twice weekly for three months. We will use low-dose vaginal estriol (1 mg/g, OeKolp Creme^®^, Dr. Kade, Berlin, Germany) administered twice weekly as maintenance after an initial short induction phase (for example, 0.5 mg daily for 7–14 days when indicated, followed by twice weekly maintenance). The control group will undergo identical follow-up procedures but receive no intervention.

The probiotic formulation consists of 10 *Lactobacillus* strains (Fido Pharma Ltd., Panchkula, India) (Table 1). Each capsule contains viable bacterial counts ranging from 2 × 10^9^ to 1 × 10^10^ colony-forming units (CFU) per strain.

### 2.4. Sampling and Follow-Up

**Figure 1:**
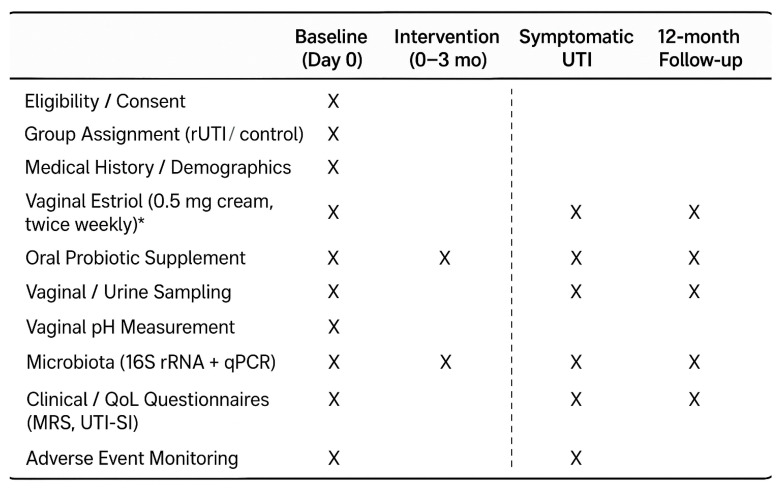
SPIRIT-style visit schedule [15]. * OeKolp^®^, Dr. Kade, Berlin, Germany. Menopause rating scale (MRS) and UTI-Symptom Impact (UTI-SI).

Biological samples will be collected at baseline, at 3 months, during any symptomatic UTI episode, and at 12 months (Figure 1).

- Vaginal samples: Collected from the mid-vaginal wall using sterile swabs and preserved in eNAT^®^ medium.
- Urine samples: Collected as midstream clean-catch specimens [3].

All samples will be stored at −80 °C until microbiological and molecular analyses are performed. We will additionally record pH immediately prior to starting estrogen and at the first follow-up after initiation to capture short-term pH change.

### 2.5. Microbiological Analyses

Urine cultures will be performed on chromogenic and CLED agar, interpreted according to EUCAST guidelines [16,17,18,19].

Presumptive bacterial identification will be based on colony morphology and color on chromogenic agar, with confirmatory identification using MALDI-TOF mass spectrometry (Bruker Daltonics, Bremen, Germany).

Antimicrobial susceptibility testing will be conducted using either the VITEK 2 automated system (bioMérieux, Saint-Denis, France) or the disk diffusion method, both according to EUCAST (European Committee on Antimicrobial Susceptibility Testing) standards [19]. Results will be reviewed by clinical microbiologists and correlated with clinical findings to ensure diagnostic accuracy.

- Recurrence: Defined as repeated positive cultures of the same pathogen with identical susceptibility profiles over time.
- Reinfection: Defined as the detection of different bacterial species or strains.

For vaginal samples, microbial DNA will be extracted using Qiagen kits with enzymatic and mechanical lysis (Singapore). The V1-V2 regions of the 16S rRNA gene will be amplified and sequenced using the Illumina MiSeq platform [20].

Bioinformatic analysis will include the following:

- Alpha diversity metrics: Shannon and Simpson indices;
- Beta diversity analysis: PERMANOVA and ordination methods;
- Differential abundance testing using R-based pipelines [21,22,23,24,25];
- Community state typing (CSTs): Determined by hierarchical clustering [26];
- Contaminant removal: Performed using the prevalence-based decontam method [27] with relative abundance (%) from 16S rRNA gene amplicon sequencing;
- Quantitative PCR (qPCR) for total bacterial 16S copies to estimate absolute bacterial load and calculate absolute *Lactobacillus* abundance.

*Lactobacillus* dominance was defined as ≥70% relative abundance or CST I/II/V. UTI was defined as ≥10^5^ CFU/mL uropathogen plus symptoms.

### 2.6. Clinical and Quality of Life (QoL) Assessments

Primary outcomes:

- Clinical diagnosis of UTI (symptoms plus positive urine culture);
- Change in *Lactobacillus* abundance;
- UTI recurrence rate.

Secondary outcomes:

- Microbial diversity indices;
- Vaginal pH;
- Quality of life (QoL) (validated questionnaire);
- Menopausal symptoms (Menopause Rating Scale, MRS) [28];
- Participant questionnaire (Table 2).

## 3. Data Collection and Statistical Analysis

Demographic and clinical data, including MRS scores and antibiotic history, will be collected using electronic case report forms (eCRFs) [28]. Statistical analyses will be performed using R software (version 4.3.1, 2023):

- Comparisons of microbial diversity and relative abundance across groups and time points: non-parametric tests (Kruskal–Wallis and Mann–Whitney U).
- Beta diversity: Assessed via PERMANOVA.
- Time-to-event analyses: Conducted using Kaplan–Meier survival curves and Cox regression.
- Multivariate logistic regression: Applied to evaluate associations between microbiota profiles and UTI recurrence.

All analyses will use a two-sided significance level of *p* < 0.05.

Timeline

▪ Study preparation: March–October 2025;
▪ Recruitment: October–December 2025;
▪ Three months follow-up completed: March 2026;
▪ Data analysis and manuscript preparation: April–May 2026;
▪ Twelve months follow-up completed: January 2027;
▪ Data analysis and submission of final report: February 2027.

## 4. Discussion

The VaMirUTI study addresses a critical gap in the management of rUTIs among menopausal women. It investigates the combined use of oral probiotics and vaginal estrogen, accompanied by detailed microbiological and clinical assessments. While previous studies have focused on probiotics or estrogen therapy separately, their potential synergistic effects have not been fully explored. Topical vaginal estrogen (estradiol or estriol) consistently increases vaginal *Lactobacillus* abundance and lowers vaginal pH in postmenopausal women. Estriol is often used for genitourinary syndrome of menopause therapy and is available in low-dose creams (0.5 mg commonly reported) with robust evidence for local effects on vaginal epithelium and microbiota and low systemic exposure.

This study incorporates several strengths. It combines comprehensive microbial and clinical evaluations, offering an integrated understanding of biological and symptomatic responses to the intervention. The longitudinal design, with multiple sampling points over 12 months, allows assessment of both short- and long-term effects on vaginal and urinary microbiota. Furthermore, the use of validated clinical instruments and robust statistical methods enhances data reliability and interpretability.

Despite these strengths, certain limitations must be acknowledged. As a single-center study, the generalizability of findings may be limited by institutional or regional factors. The lack of a placebo control group could introduce subjective bias, especially in patient-reported outcomes. Moreover, interindividual variability in microbiota composition may affect the reproducibility, potentially diluting observed effects. Finally, although the intervention lasts three months, this duration may be insufficient to induce lasting microbial or clinical changes, emphasizing the need for extended follow-up in future studies.

Although this study is observational, its findings will help refine eligibility criteria, endpoint selection, and effect-size estimates for a future randomized controlled trial comparing estriol with and without probiotic supplementation.

## Figures and Tables

**Table 1:** Composition of the probiotic formulation and viable counts per capsule.

Probiotic Strain	Strain ID	Viable Count (CFU *)
*Limosilactobacillus reuteri*	FPPL-109004	3 × 10^9^ (3 billion)
*Lactiplantibacillus plantarum*	FPPL-109005	4 × 10^9^ (4 billion)
*Lactobacillus crispatus*	FPPL-109016	3 × 10^9^ (3 billion)
*Lactobacillus gasseri*	FPPL-109019	2 × 10^9^ (2 billion)
*Lactobacillus jensenii*	FPPL-109014	1 × 10^10^ (10 billion)
*Ligilactobacillus salivarius*	FPPL-109009	1 × 10^10^ (10 billion)
*Lacticaseibacillus rhamnosus*	MTCC-25742	1 × 10^10^ (10 billion)
*Lacticaseibacillus paracasei*	MTCC-25843	3 × 10^9^ (3 billion)
*Lacticaseibacillus casei*	FPPL-109003	2 × 10^9^ (2 billion)
*Lactobacillus acidophilus*	FPPL-109010	3 × 10^9^ (3 billion)

* CFU = colony-forming units per capsule.

**Table 2:** Participant questionnaire at study start and completion.

Domain	Study Start	End of Study
General well-being	How would you rate your general well-being?	How would you rate your general well-being now?
Burden of condition	How much does your condition burden you?	How much does your condition burden you now?
Daily life limitation	How limited are you in your daily life?	How limited are you in your daily life now?
Study expectations	How high are your expectations regarding participation in the study?	Were your expectations fulfilled?
Treatment success	–	How do you assess the success of the treatment?

Each item is rated on a 5-point Likert scale ranging from very poor/none to very good/completely.

## Data Availability

The detailed data presented in this study are available on request from the corresponding author due to practicability.
